# L-arginine Results in an Artificial Increase in Creatinine

**DOI:** 10.7759/cureus.93866

**Published:** 2025-10-05

**Authors:** Michelle Hwang, Jennifer Han, Sean Lei, Igor Kagan

**Affiliations:** 1 Medicine, Ronald Reagan University of California Los Angeles Medical Center, Los Angeles, USA; 2 Endocrinology, Diabetes and Metabolism, University of California Los Angeles David Geffen School of Medicine, Los Angeles, USA; 3 Nephrology, Ronald Reagan University of California Los Angeles Medical Center, Los Angeles, USA

**Keywords:** artificial increase in creatinine, cystatin c, increase in creatinine, l-arginine, supplement

## Abstract

L-arginine is a commonly available over-the-counter supplement. We report a 65-year-old man with a history of benign prostatic hyperplasia, hypertension, and obstructive sleep apnea who had an elevated serum creatinine level and normal cystatin C in the setting of L-arginine supplementation. After discontinuation of L-arginine, repeat testing showed normalization of serum creatinine, while cystatin C remained unchanged, thus demonstrating that L-arginine use can result in an artificial increase in serum creatinine.

## Introduction

L-arginine is a semi-essential amino acid [1] commonly available as an over-the-counter supplement. It is commonly believed that L-arginine can improve exercise tolerance, erectile dysfunction [2], and nitric oxide synthesis. Because of these properties, it is at times used by active individuals to enhance their performance. Studies in the past have shown that administration of L-arginine can result in increased creatinine levels [3,4]. With the recent adaptation of additional blood tests, such as cystatin C, that do not rely on creatinine as a source of kidney function measurement, we are able to show that L-arginine results in an artificial increase in creatinine.

## Case presentation

A 65-year-old physically active male was referred to the nephrology clinic for evaluation of an elevated serum creatinine on routine laboratory testing. He has a past medical history of benign prostatic hyperplasia, hypertension, and obstructive sleep apnea. His medications included amlodipine 5 mg daily and losartan 100 mg daily. Losartan was increased from 50 mg to 100 mg daily about five months prior. He denied nonsteroidal anti-inflammatory drugs (NSAIDs) but reported taking over-the-counter L-arginine supplements (1,000 mg daily) for the past three to four years for general health. On exam, the patient was a healthy 65-year-old male without any obvious physical findings.

Laboratory results were reviewed, and his serum creatinine was 1.33 mg/dL one year prior to his appointment, with the normal reference range for creatinine of 0.60-1.30 mg/dL (Table 1). Another lab test five months prior to his nephrology appointment showed a serum creatinine of 1.43 mg/dL, which was repeated two months later by primary care, showing a creatinine of 1.68 mg/dL and a cystatin C of 0.8 mg/L on the same blood draw. Prior urinalysis were normal, showing no proteinuria or microscopic hematuria (Table 2).

**Table 1:** Metabolic parameters and kidney function tests before and after L-arginine supplementation

Test	Reference range	Baseline levels (1 year prior)	On L-arginine supplementation	On L-arginine supplementation	1 week after the discontinuation of L-arginine supplementation	7 months after the discontinuation of L-arginine supplementation
Creatinine	0.60-1.30 mg/dL	1.33 mg/dL	1.43 mg/dL	1.68 mg/dL	1.16 mg/dL	1.2 mg/dL
Cystatin C	0.50-1.20 mg/L		-	0.8 mg/L	0.9 mg/L	0.9 mg/L

**Table 2:** Urinalysis prior and one week after the discontinuation of L-arginine supplementation

Variables	Reference range	Prior L-arginine supplementation	1 week after the discontinuation of L-arginine supplementation
Blood, Dipstick	Negative	Negative	Negative
Protein	Negative	Negative	Negative
RBC per uL	0-11 cells/uL	3	1

**Figure 1:**
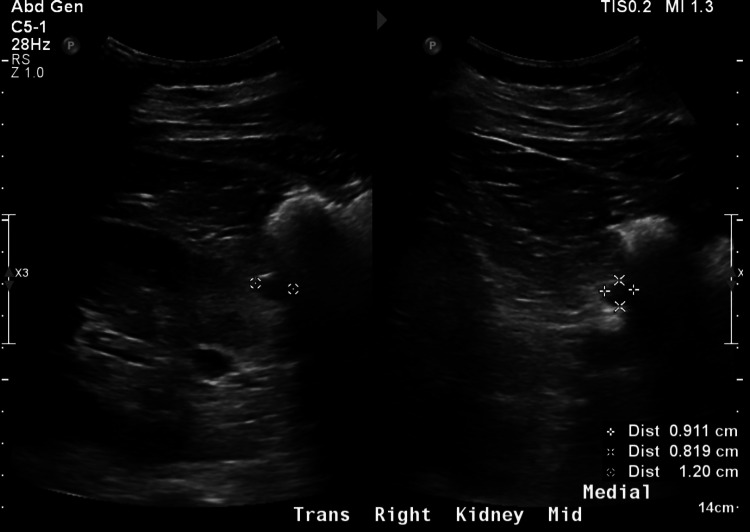
Kidney ultrasound reveals a simple-appearing cyst in the midpole of the right kidney, measuring 1.2 cm

A prior renal ultrasound showed normal-sized kidneys, with normal cortical thickness and echogenicity, and no hydronephrosis. There was a simple appearing right renal cyst measuring 1.2 cm in the midpole (Figure 1).

After a full history and physical during the nephrology consultation, it was determined that L-arginine may be the cause of the increase in creatinine. Patient agreed to hold L-arginine supplements for one week and repeat a metabolic panel along with cystatin C. On the repeat metabolic panel after not taking L-arginine, serum creatinine was 1.16 mg/dL, and cystatin C was similar to prior at 0.9 mg/L. A repeat urinalysis showed no changes, with no evidence of proteinuria or microscopic hematuria. Losartan dose-related creatinine increase and hypertension-related CKD progression are unlikely contributors in this case because they would also result in an increase in cystatin C, which had been stable. The patient continued to hold off taking L-arginine and had bloodwork repeated seven months later, which showed a normal creatinine at 1.2 mg/dL.

## Discussion

Amino acids are a widely used nutritional supplement and are often used by athletes to improve performance [5]. L-arginine is a semi-essential amino acid that has been used to enhance performance by stimulating growth hormone secretion and post-exercise muscle recovery, decreasing lactate levels, and increasing cardiac function [5,6]. With the advent of new laboratory testing, such as cystatin C, we were able to document that L-arginine supplementation can result in an artificial increase in serum creatinine. In this case, the patient had an elevated serum creatinine while he was taking L-arginine supplements, yet had a normal cystatin C result. After discontinuing L-arginine, repeat blood work one week later showed normalization of serum creatinine, with cystatin C remaining unchanged.

The likely mechanism behind the elevated creatinine is related to increased synthesis of creatine. It is well known that creatine supplementation increases the serum creatinine levels [7]; however, L-arginine is also in the metabolic pathway for creatine creation. L-arginine is the initial substrate that combines with glycine to form guanidinoacetate (GAA), the rate-limiting step in creatine synthesis. GAA is then methylated to form creatine, which is subsequently metabolized in muscle tissue and non-enzymatically converted to creatinine [8]. The link between L-arginine supplementation and increased creatinine production is supported by animal studies in which arginine infusion increased GAA, the precursor of creatinine [9]. We hypothesize that the oral intake of L-arginine enhances this pathway, resulting in an increase in creatine, which then is detected on laboratory blood work as an increased serum creatinine level. However, cystatin C is not affected by this metabolic pathway, explaining the observed discrepancy between the elevated creatinine and normal cystatin C levels in this patient.

This case highlights the important role of cystatin C as an alternative biomarker. Unlike creatinine, cystatin C is not significantly affected by muscle mass or amino acid supplementation and thus provides a more reliable estimate of kidney function when creatinine values appear discordant. Current guidelines increasingly recommend the use of cystatin C, particularly when the creatinine-based estimated glomerular filtration rate (eGFR) is inconsistent with the clinical picture [10]. In our case, cystatin C helped rule out kidney disease and prevented unnecessary investigations.

More broadly, our case emphasizes the importance of reviewing non-prescription medication use, including over-the-counter supplements. Such products are easily accessible and often go unreported, yet usage can significantly affect laboratory results, potentially leading to unnecessary evaluations, including imaging or even biopsy. Recognizing that supplements such as L-arginine can produce spurious creatinine elevations may help clinicians avoid excessive testing, reduce patient anxiety, and lower healthcare costs.

## Conclusions

This case highlights the importance of continued development and use of new laboratory tests to better evaluate kidney function and the importance of considering a wide differential diagnosis, especially when seeing routine patients with elevated serum creatinine. In this case, cystatin C testing provided reassurance to both the patient and the clinician by confirming that underlying renal function remained within the normal range. Clinicians should remember to inquire about over-the-counter supplements as such information can be critical in reaching an accurate diagnosis.
